# Supplementary material for: Empirical methods for controlling false positives and estimating confidence in ChIP-Seq peaks
Source: BMC Bioinformatics. 2008 Dec 5;9:523. doi: 10.1186/1471-2105-9-523 (PMC2628906; doi:10.1186/1471-2105-9-523)
Supplement: Additional File 1 — A variety of html documents from the USeq web site detailing the available applications, their best usage, output file type descriptions, command line menus, etc. [file 1471-2105-9-523-S1.zip › USeqUserGuides/JavaDoc.zip!/JavaDoc/trans/anno/package-use.html]

Uses of Package trans.anno


|  |  |  |
| --- | --- | --- |
| **Overview** \| **Package** \| Class \| **Use** \| **Tree** \| **Deprecated** \| **Index** \| **Help** | |  |
| PREV   NEXT | **FRAMES**    **NO FRAMES**     **All Classes** |  |


---


## **Uses of Package trans.anno**

| Packages that use trans.anno | |
| --- | --- |
| **trans.anno** | For associating intervals, binding regions, and binding peaks with GFF annotation. |
| **util.bio.parsers** | Parsers and extractors, fasta, quality, text, gff.... |

Classes in trans.anno used by trans.anno

****BindingRegion****
  
          For holding information about a genomic interval, what genes are up, down, overlap, or contain this genomic inteval.

****RandomRegions****
  
          Use to generate random regions of a defined length from a file of regions (chrom, start, stop).

****Region****
  
          Represents a genomic region.

Classes in trans.anno used by util.bio.parsers

****BindingRegion****
  
          For holding information about a genomic interval, what genes are up, down, overlap, or contain this genomic inteval.

---


|  |  |  |
| --- | --- | --- |
| **Overview** \| **Package** \| Class \| **Use** \| **Tree** \| **Deprecated** \| **Index** \| **Help** | |  |
| PREV   NEXT | **FRAMES**    **NO FRAMES**     **All Classes** |  |


---
